# Genetic Testing and/or Counseling for Colorectal Cancer by Health Insurance Type

**DOI:** 10.3390/jpm12071146

**Published:** 2022-07-15

**Authors:** Arian Mansur, Fang Zhang, Christine Y. Lu

**Affiliations:** 1 Harvard Medical School, Boston, MA 02115, USA; arianmansur@hms.harvard.edu; 2 Department of Population Medicine, Harvard Medical School and Harvard Pilgrim Health Care Institute, 401 Park Drive, Suite 401 East, Boston, MA 02215, USA; fang_zhang@harvardpilgrim.org

**Keywords:** genetic testing, genetic counseling, colorectal cancer, health insurance, genomic medicine, precision medicine

## Abstract

Genetic testing is increasingly used in clinical practice to assist with the diagnosis of genetic diseases and/or provide information about disease risk, and genetic counseling supports patient understanding of test results before and/or after genetic testing. Therefore, access to genetic testing and counseling is important for patient care. Health insurance coverage is a major determinant of access to health care in the United States. Uninsured individuals are less likely to have a regular source of health care than their insured counterparts. Different health insurance types and benefits also influence access to health care. Data on the association of health insurance and uptake of genetic testing and/or counseling for cancer risk are limited. Using data from the National Health Interview Survey, we examined the uptake of genetic testing and/or counseling for colorectal cancer (CRC) risk by health insurance type. We found that only a small proportion of individuals undergo genetic testing and/or counseling for CRC risk (0.8%), even among subgroups of individuals at risk due to family or personal history (3.7%). Insured individuals were more likely to undergo genetic testing and/or counseling for CRC risk, particularly those with Military and Private insurance plans, after adjusting for various demographic, socioeconomic, and health risk covariates. Further investigations are warranted to examine potential disparities in access and health inequities.

## 1. Introduction

Genomic medicine, the interdisciplinary field that involves the use of patients’ genomic information to guide clinical care, has rapidly expanded since the completion of the Human Genome Project [1]. With the accelerated advances in technology, genetic testing and counseling are now becoming much more readily accessible than they were decades ago, and have a wide range of uses from diagnostic and predictive purposes to the examination of one’s ancestry [2]. One such purpose of genetic testing that has proven to be clinically promising is the detection of an individual’s risk of colorectal cancer (CRC), which is the second leading cause of cancer mortality in the United States with approximately 30% of cases believed to have a familial component, and about 5–10% are hereditary [3,4,5,6].

While genetic testing can help assess individuals’ risk of cancer and guide screening and preventive measures, access to genetic testing and counseling may be influenced by individuals’ health insurance in the United States. The United States Health system is a mix of public and private, for-profit and nonprofit insurers [7]. The national Medicare program covers care for adults aged 65 and older and some people with disabilities. The federal government also provides funding for various programs for veterans and low-income people, including Medicaid and the Children’s Health Insurance Program, with the states managing and paying for local coverage. Private insurance is the dominant form of insurance for nonelderly adults and is provided primarily by employers. It is estimated that 8.5% of the population is uninsured, an improvement from 16% in 2020 [7]. There are wide variations in insurance coverage of genetic services. While most plans will cover testing that is recommended by a physician, the exact policies on coverage and reimbursements depend on the type of health insurance, and costs varies between several hundreds to thousands USD per test [8,9]. Coverage policies are not available for all forms of genetic testing, and existing ones are inconsistent across insurers with a lack of transparency [10]. For instance, it is often a challenge for insurers to know when to reimburse for genetic services as they are often not able to identify which ones are administered [11]. This is challenging because these tests are billed according to the Current Procedural Terminology (CPT) standardized codes developed by the American Medical Association that have less than 200 codes for ~70,000 genetic tests [11]. Genetic discrimination is also another concern with regard to the intersection of genomic medicine and insurance, whereby individuals often worry that their genetic results will be adversely used to deny coverage or determine premiums, despite the Genetic Information Non-discrimination Act (GINA) prohibiting such discrimination by employers and health insurance companies [12].

While it is known that health system (e.g., health insurance) levels, insurance coverage policies, and the degree of patient cost-sharing affects patient access to genomic testing [13,14,15,16,17,18], there is a lack of data specific to CRC. The objective of this study was to examine the association between the uptake of genetic counseling and/or genetic testing for CRC and type of health insurance using the National Health Interview Survey (NHIS).

## 2. Materials and Methods

### 2.1. Data Source

Data was extracted from the publicly available Integrated Public Use Microdata Series National Health Interview Survey (NHIS) project, which collects census data from the NHIS [19]. The NHIS is an annual survey conducted by the U.S. Census Bureau on behalf of the National Center of Health Statistics, which is part of the U.S. Centers for Disease Control and Prevention. The NHIS constitutes the primary source of data on the health status and health care access of the civilian noninstitutional population and has been instrumental in monitoring progress towards national health objectives [20].

We limited our analysis to 2015 since that was the latest Cancer Control Supplement, which contains data on individual genetic testing behavior, available with CRC data at the time of the study. 

### 2.2. Data Measures

Our main outcome variable assessed genetic testing and/or genetic counseling for CRC risk. Our control group were those who answered “No” to both survey questions: “*Have you EVER HAD a genetic test to determine if you are at greater risk of developing cancer in the FUTURE? This does not include any test to see whether you had cancer in the PAST or have cancer NOW.*” and “*These next questions refer to genetic COUNSELING for cancer risk. We will ask about genetic TESTING for cancer risk in a few minutes. Genetic counseling involves a discussion with a specially trained health care provider about your family history of cancer and how likely you are to develop cancer. It may also include a discussion about whether genetic testing is right for you. Have you ever received genetic counseling for cancer risk?*” Those with genetic testing and/or genetic counseling for CRC risk were those who answered “Yes” to above-mentioned survey questions, in addition to answering “Yes” to either question specific to CRC: “*Please think about your MOST RECENT genetic test for cancer risk. Was it for colon or rectal cancer?*” and “*Please think about your MOST RECENT genetic counseling session for cancer risk. Was it for colon or rectal cancer?*” 

Our primary predictor of interest was health insurance type (no insurance, Medicaid, Medicare, Military, Dual [Medicaid and Medicare], Other Public, and Private). ‘Military’ includes health insurance coverage through some form of military health insurance, e.g., Veteran Affairs (VA) health insurance, and ‘Other Public’ includes health care coverage provided by a public program other than Medicare, Medicaid, Military (e.g., state-sponsored health insurance). In our preliminary analysis, there were no meaningful differences between Private health insurance with or without high deductible (possibly due to sample size) and therefore we created only one group of individuals with any Private insurance. Individuals with unknown health insurance were excluded from the analysis. 

Demographic characteristics included age, sex, race, marital status, education level, and income level. Other predictors of interest included personal history of CRC, family history of CRC, self-perceived CRC, and chronic conditions. Self-perceived risk was assessed by participants’ response to the question “Compared to the average [fill1: man/woman] your age, would you say that you are more likely to get colon or rectal cancer, less likely, or about as likely? For a colon or rectal cancer survivor, this means getting colon or rectal cancer again in the future.” Response options included “More likely”, “Less likely”, “About as likely”, or “Don’t know”. Chronic conditions was defined as having had at least one of the various following conditions: hypertension, coronary heart disease, diabetes, cancer (not including CRC), stroke, chronic bronchitis, emphysema, current asthma, and kidney disease [21]. 

### 2.3. Data Analysis

Given the complex survey design of the NHIS, statistical analyses were adjusted with sampling weights and variance estimation methodologies using the survey module in StataMP, version 17.0 for Mac (StataCorp, College Station, TX, USA). Descriptive statistics were created for weighted samples. Comparisons between those with versus those without genetic testing and/or counseling were evaluated using Student’s t test or Wilcoxon rank sum test for continuous variables and Pearson’s χ^2^ test or Fisher’s exact test for categorical variables. Simple and multivariable logistic regression models were generated to examine the association between genetic testing and/or counseling for CRC and health insurance type. This analysis was repeated in a subgroup of ‘at-risk’ individuals with either a personal history of CRC, family history of CRC, or believed that their risk of CRC was more likely when compared to an average person of same age (*n* = 3191). 

## 3. Results

**Figure 1:**
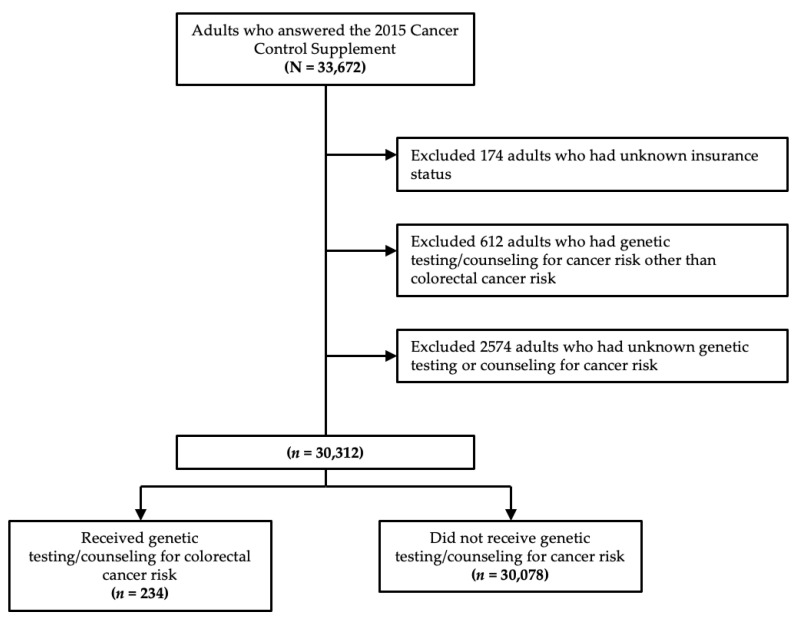
Study flow diagram.

Of 30,312 individuals that met the study criteria, 234 people (0.8% weighted) received genetic testing and/or counseling for CRC risk (Figure 1). Among 3191 at-risk individuals, 109 people (3.7% weighted) received genetic testing and/or counseling for CRC risk. Among 211 at-risk individuals who had a personal history of CRC, 15 (8.4% weighted) got genetic testing and/or counseling for CRC risk. Table 1 summarizes the baseline characteristics of our study cohort. Those who received genetic testing/counseling for CRC risk were older, more likely to have gone to college, more likely to have a personal and family history of CRC, viewed themselves as more likely to get CRC compared to an average person of the same age, were more likely to have at least one chronic condition, and were more likely to have some form of health insurance (Table 1). 

Table 2 shows results from the univariable and multivariable logistic regression models estimating the association between genetic testing and/or counseling for CRC risk and health insurance type. In unadjusted analysis, individuals across all health insurance types were significantly more likely to have had uptake of genetic counseling and/or testing for CRC risk than those without health insurance. In adjusted analysis, individuals with Medicaid, Military, Other Public, and Private health insurance plans were significantly more likely to have had genetic counseling and/or testing for CRC risk than those without any health insurance.

Among the subgroup of individuals who had either a personal history of CRC, family history of CRC, or believed that their risk of CRC was more likely than an average person of same age, the unadjusted analysis found that individuals with Military and Private health insurance types were significantly more likely to have had an uptake of genetic counseling and/or testing for CRC risk than those without health insurance (Table 3). In adjusted analysis, individuals with Military and Private health insurance plans were significantly more likely to have had genetic counseling and/or testing for CRC risk than those without any health insurance (Table 3).

## 4. Discussion

In this national analysis of adult self-reported survey data, we found that only a small proportion of individuals undergo genetic testing and/or counseling for CRC risk, even among subgroups of individuals at risk due to family or personal history (3.7%) and those at risk due to personal history of CRC (8.4%). Similarly, Actkins et al. also found very low uptake of genetic testing in adults who had CRC and/or endometrial cancer using NHIS survey data [22]. While genetic testing/counseling is available for inherited cancer syndromes [23], such as Lynch syndrome or familial adenomatous polyposis, most CRC is not caused by inherited mutations; this may explain our finding of a low rate of genetic testing/counseling for CRC risk. However, universal screening for DNA mismatch repair deficiency is recommended by the American Society of Clinical Oncology (ASCO) and the European Society for Medical Oncology (ESMO) among all newly diagnosed CRC patients [24]. Furthermore, genetic testing and/or counseling are recommended for individuals who have family members with inherited cancer syndromes [23,25,26]. Specifically, ASCO recommends that genetic testing for mutations that cause known cancer susceptibility syndromes should be offered when three criteria are met: the presence of a personal or family history suggestive of genetic cancer susceptibility; the genetic test can be adequately interpreted; and the test results have clinical utility in diagnosing or influencing subsequent management of the patient or family members at hereditary risk of cancer [27,28]. However, ASCO recently suggested that providers with a particular expertise in cancer risk assessment should be involved in ordering multigene testing, such as panel testing, which may include genes associated with a moderate or low risk for cancer and high-penetrance genes that would not otherwise be evaluated on the patient’s personal or family history. Nonetheless, ASCO affirms that it is sufficient for cancer risk assessment to evaluate genes of established clinical utility on the basis of a patient’s personal and/or family history [29]. Our study and others suggest that efforts are still needed to increase genetic testing/counseling for improving treatment and prevention of CRC and public health.

We also identified an association between genetic testing and/or counseling for CRC risk and type of health insurance, adjusting for various demographic, socioeconomic, and health risk covariates. We found that individuals with Medicaid, Military, Other Public, and Private health insurance plans were significantly more likely to receive genetic testing and/or counseling for CRC risk than those with no health insurance. Importantly, in a subgroup analysis limited to at-risk patients for CRC, we also found that receipt of genetic testing and/or counseling differed by health insurance type; people with Military and Private health insurance were significantly more likely to have had genetic testing and/or counseling relative to those with no health insurance. The statistical insignificant results for some of the health insurance types in the multivariable regression models were likely due to the lower rates of uptake after adjusting for covariates. While it is reassuring that some people with health insurance (particularly those at-risk and would benefit from access) have greater access to genetic testing/counseling for CRC risk than the uninsured, our findings suggest access likely differed across insurance types. The complexities in health insurance coverage and variations in access to health services in the US are well known [30,31,32,33]. Similar complexity and variations in access also extend to genetic services. Health insurance coverage policies do not exist for all types of genetic testing. Further, there are substantial variations in existing coverage policies across insurers for guideline-recommended pharmacogenetic tests as well as for genetic tests for identification of hereditary cancer risk [18,34]. There is increasing evidence that supports the use of germline cancer tests in patients being evaluated for hereditary cancer [35,36]. Such results can inform cancer screening recommendations and surgical considerations. Research [10] suggests that health insurance coverage policies may not meaningfully differentiate between patients with cancer who are likely versus unlikely to benefit from germline genetic testing for cancer. It is imperative that health insurance coverage policies support prevention to reduce the number of cancer cases, especially in hereditary cancers where genetic tests are recommended in individuals that meet clinical criteria. This genetic testing and subsequent management not only prevent cases of cancer in families harboring the mutation(s) but also improve treatment and personalized medicine through the acquired knowledge of mutations. It is also important to note that these health benefits extend to everyone, regardless of health insurance status, as health spending savings are a consequence of health prevention [37]. 

There have been other studies using the NHIS to study the utilization of genetic testing and/or counseling [22,38,39,40]. Actkins et al. examined the uptake of genetic testing across 2005, 2010, and 2015 in adults who had CRC and/or endometrial cancer [22], and found that uninsured individuals had higher rates of genetic testing [22]. This finding is counterintuitive and conflicts with ours. It is worth noting that they focused on a different population of interest, their definition of genetic testing encompassed other cancer risks, and their health insurance variable did not account for differences in insurance type as insurance was not the focus of their work. In another study, Turbitt et al. examined genetic testing and counseling for CRC or breast cancer risk among people with no personal cancer history and found that having insurance was associated with undergoing genetic counseling for CRC risk but not with genetic testing [38]. Again, these results may not be fully comparable to ours because they excluded individuals under 50 years of age and those with a personal history of CRC, and they did not use an expanded version of insurance type [38]. Stamp et al. analyzed genetic counseling using the NHIS data and stratified their health insurance variable into different types [40]. They found that while insurance status was associated with genetic counseling, it was not so after covariate adjustment [40]. Interestingly, their reference group for insurance was Private insurance while ours was Uninsured. They also say that in their unaffected cohort, insurance was not significant, but health insurance type (“Other”) shows significant increase in genetic counseling when compared to Private (*P* = 0.02). Furthermore, they focused on genetic counseling for multiple cancer risks while we focused mainly on CRC risk, and they stratified their results based on personal history of cancer [40]. The heterogeneity in these studies (and their methods) makes the comparison between findings difficult. 

There are several strengths to our study. It is one of the first to systematically analyze the various types of health insurance and their association with uptake of genetic services for CRC. Furthermore, by using the NHIS, we were able to create a larger cohort representative of the national population to not only study the effects on a specific cancer but also limit confounders. Nonetheless, there are several limitations to the present study. First, as a cross-sectional analysis, we cannot make claims about temporal relationships between insurance types and uptake of genetic testing and/or counseling for CRC. Furthermore, as an observation study, we cannot make any causal claims and there is the possibility of confounding variables which were not accounted for. We attempted to minimize this bias by performing a subgroup analysis limited to patients who were at-risk for CRC (a more homogeneous group) and for whom health insurance policies are likely to be similar. While the NHIS collects some information about patient cost-sharing, we were unable to examine its interactive effects with health insurance types due to small sample sizes. Our variables were limited by the design of the NHIS survey. For instance, we were unable to identify people who have family members with inherited cancer syndromes (who might benefit from genetic testing/counseling based on clinical guidelines) thus our at-risk subgroup included individuals with family history of CRC. Furthermore, NHIS survey does not have information on specific types of CRC risk (e.g., polyposis, juvenile polyposis syndrome, hereditary nonpolyposis colorectal cancer, lynch syndrome), which may influence genetic testing and counseling decisions for the individual and his/her family members including children (in those with hereditary polyposis) [41]. Finally, self-reported survey data may be vulnerable to recall bias [42].

## 5. Conclusions

Our findings suggest variations in patient access to genetic testing and/or counseling for CRC risk across health insurance types. Currently, a lack of standardization in health insurance coverage policies for genetic testing/counseling may be a barrier for patients who could benefit from such services. Further investigations are warranted to examine potential disparities in access and health inequities. Efforts to minimizing variations in health insurance coverage policies across insurers for genetic testing/counseling and aligning coverage policies with professional society guidelines are needed for equitable integration of genetic services into routine clinical care.

## Figures and Tables

**Table 1:** Baseline Characteristics.

Variable	Received Genetic Testing/Counseling	Did Not Receive Genetic Testing/Counseling	p *
Unweighted N (weighted %)	234 (0.8)	30,078 (99.2)	
Insurance Type, unweighted No. (weighted %)			<0.01
Uninsured	10 (2.7)	3257 (9.7)	
Medicaid	23 (9.0)	2891 (8.6)	
Medicare	47 (21.9)	6484 (21.6)	
Military	27 (10.3)	1435 (4.6)	
Dual	16 (5.6)	1004 (3.0)	
Other Public	4 (2.2)	280 (0.9)	
Private	107 (48.3)	14,727 (51.7)	
Age (years), weighted mean (SE)	55.8 (1.26)	49.4 (0.21)	<0.01
Sex, unweighted No. (weighted %)			0.26
Male	116 (50.8)	13,641 (46.4)	
Female	118 (49.2)	16,437 (53.6)	
Race, unweighted No. (weighted %)			0.09
White	170 (75.1)	23,245 (80.1)	
Black	46 (17.5)	4007 (12.2)	
Other	18 (7.4)	2826 (7.8)	
Marital Status, unweighted No. (weighted %)			0.48
Married/live with partner	121 (53.3)	15,136 (50.2)	
Not currently married ^1^	113 (46.7)	14,891 (49.6)	
Unknown	0 (0.0)	51 (0.2)	
Education, unweighted No. (weighted %)			0.03
Less than college	66 (27.3)	11,681 (36.0)	
College	167 (72.4)	18,288 (63.7)	
Unknown	1 (0.3)	109 (0.4)	
Combined Family income, Unweighted No. (weighted %)			0.19
At or above poverty line	187 (81.7)	24,210 (82.0)	
Below poverty line	43 (17.3)	4512 (13.6)	
Unknown	4 (1.0)	1356 (4.4)	
Personal History of CRC, unweighted No. (weighted %)			<0.01
Yes	15 (7.7)	196 (0.7)	
No	219 (92.3)	29,882 (99.3)	
Family History of CRC, unweighted No. (weighted %)			<0.01
Yes	74 (32.3)	1678 (5.6)	
No	160 (67.7)	28,400 (94.4)	
Self-perceived CRC risk, unweighted No. (weighted %)			<0.01
Less likely	61 (26.0)	12,772 (42.0)	
About as likely	96 (41.9)	13,417 (45.1)	
More likely	67 (27.5)	1773 (5.9)	
Unknown	10 (4.6)	2116 (7.1)	
Chronic conditions, unweighted No. (weighted %)			<0.01
None	81 (36.2)	15,235 (51.5)	
At least 1	153 (63.8)	14,793 (48.4)	
Unknown	0 (0)	50 (0.2)	

Abbreviations: CRC, colorectal cancer; No., number; SE, standard error. ^1^ Included people who are widowed, divorced, separated, or never married. * *p*-values were adjusted for sampling weights.

**Table 2:** Logistic Regression Evaluating Association Between Uptake of Genetic Testing/Counseling for Colorectal Cancer and Health Insurance Type.

Variable	OR (95% CI)	*p*	aOR (95% CI)	*p*
Insurance type (ref = uninsured)				
Medicaid	**3.69 (1.44–9.44)**	**<0.01**	**2.88 (1.10–7.53)**	**0.03**
Medicare	**3.59 (1.51–8.52)**	**<0.01**	1.47 (0.57–3.79)	0.42
Military	**7.96 (3.19–19.82)**	**<0.01**	**3.54 (1.32–9.50)**	**0.01**
Dual	**6.75 (2.56–17.80)**	**<0.01**	2.18 (0.79–6.03)	0.13
Other Public	**8.52 (1.81–40.12)**	**<0.01**	**6.64 (1.25–35.3)**	**0.03**
Private	**3.30 (1.46–7.44)**	**<0.01**	**2.91 (1.26–6.72)**	**0.01**
Age (per year)	–	–	1.02 (1.00–1.03)	0.01
Female versus male	–	–	0.80 (0.56–1.13)	0.21
Race (ref = white)				
Black	–	–	1.95 (1.26–3.04)	<0.01
Other	–	–	1.24 (0.70–2.19)	0.46
Married/live with partner versus not currently married ^1^	–	–	0.87 (0.59–1.29)	0.49
Education less than college versus college	–	–	1.66 (1.11–2.48)	0.01
Household Income at or above versus below poverty line	–	–	1.76 (1.11–2.79)	0.02
No versus at least one chronic condition			1.33 (0.85–2.09)	0.21
Personal history of CRC versus no history	–	–	4.98 (2.53–9.82)	<0.01
Family history of CRC versus no history	–	–	4.81 (3.10–7.44)	<0.01
Perceived cancer risk in self (ref = less likely)				
About as likely	–	–	1.47 (1.00–2.15)	0.05
More likely	–	–	3.85 (2.36–6.29)	<0.01

Abbreviations: CRC, colorectal cancer; ^1^ Included people who are widowed, divorced, separated, or never married. Bold indicates statistical significance (*p* < 0.05) in the insurance type variable.

**Table 3:** Logistic Regression Evaluating Association Between Uptake of Genetic Testing/Counseling for Colorectal Cancer and Health Insurance Type for At-Risk People.

Variable	OR (95% CI)	*p*	aOR (95% CI)	*p*
Insurance type (ref = uninsured)				
Medicaid	3.63 (0.92–14.33)	0.07	3.20 (0.79–12.90)	0.10
Medicare	2.73 (0.89–8.39)	0.08	2.43 (0.79–7.44)	0.12
Military	**5.33 (1.31–21.66)**	**0.02**	**4.80 (1.17–19.71)**	**0.03**
Dual	2.08 (0.46–9.42)	0.34	1.64 (0.33–8.16)	0.55
Other Public	2.18 (0.31-15.15)	0.43	2.98 (0.41–21.44)	0.28
Private	**3.95 (1.27–12.32)**	**0.02**	**3.66 (1.16–11.48)**	**0.03**
Age (per year)	–	–	1.01 (0.99–1.03)	0.32
Female versus male	–	–	1.39 (0.85–2.26)	0.19
Race (ref = white)				
Black	–	–	1.22 (0.70–2.14)	0.49
Other	–	–	1.59 (0.74–3.42)	0.24
Married/live with partner versus not currently married ^1^	–	–	0.67 (0.40–1.12)	0.12
Education less than college versus college	–	–	1.52 (0.89–2.57)	0.12
Household Income at or above versus below poverty line	–	–	1.61 (0.71–3.62)	0.25
No versus at least one chronic condition			0.85 (0.47–1.54)	0.59

^1^ Included people who are widowed, divorced, separated, or never married. Bold indicates statistical significance *(p* < 0.05) in the insurance type variable.

## Data Availability

The data presented in this study are publicly available and can be downloaded from https://www.cdc.gov/nchs/nhis/nhis_2015_data_release.htm, accessed on 15 November 2021.
